# Prognostic value of systemic immune-inflammation index in patients with small-cell lung cancer treated with immune checkpoint inhibitors

**DOI:** 10.1186/s12885-025-13440-5

**Published:** 2025-01-06

**Authors:** Yusuke Hamakawa, Ayumi Hirahara, Akiko Hayashi, Kota Ito, Hiroyuki Shinohara, Aya Shiba, Yuko Higashi, Masaharu Aga, Kazuhito Miyazaki, Yuri Taniguchi, Yuki Misumi, Yoko Agemi, Yukiko Nakamura, Tsuneo Shimokawa, Hiroaki Okamoto

**Affiliations:** Department of Respiratory Medicine and Oncology, Yokohama Municipal Citizen’s Hospital, 1-1, Mitsuzawa Nishimachi, Kanagawa Ku, Yokohama, 221-0855 Japan

**Keywords:** Small-cell lung cancer, Immune checkpoint inhibitor, Systemic immune-inflammation index, Neutrophil-to-lymphocyte ratio, Prognostic biomarker

## Abstract

**Introduction:**

The systemic immune-inflammation index (SII) has emerged as a promising prognostic marker in various malignancies. However, its prognostic significance in patients with small-cell lung cancer (SCLC) treated with immune checkpoint inhibitors (ICIs) remains unclear. In this study, we evaluated the prognostic impact of the SII in patients with SCLC after ICI use.

**Methods:**

Of 62 patients with SCLC who received chemoimmunotherapy at our institution between September 2019 and July 2024, we retrospectively analyzed 36 patients who subsequently received ICI maintenance therapy following the initial chemoimmunotherapy treatment. The SII was calculated at the start of the second cycle of the ICI maintenance therapy. Patients were stratified into high (≥ 570) and low (< 570) SII groups. Overall survival (OS) and progression-free survival (PFS) were compared between the groups using the Kaplan–Meier method and log-rank test. Multivariate analysis using the Cox proportional hazards model was performed to identify independent prognostic factors.

**Results:**

The high SII group exhibited a significantly shorter OS (median 12.1 vs. 24.1 months, *P* = 0.010) and PFS (median 5.2 vs. 8.1 months, *P* = 0.026) than those in the low SII group. A multivariate analysis identified SII ≥ 570 as an independent negative prognostic factor for OS (hazard ratio 3.83, 95% confidence interval 1.38–10.6, *P* = 0.010).

**Conclusions:**

Elevated SII in the initial phase of ICI maintenance therapy was associated a with poor prognosis in patients with SCLC, supporting its utility as a prognostic biomarker in this setting. Therefore, prospective validation is required to confirm these findings.

## Introduction

Inflammation influences tumor progression through the secretion of bioactive molecules into the tumor microenvironment. Inflammatory mediators promote key tumor characteristics including proliferation, survival, angiogenesis, invasion, and metastasis. Consequently, inflammation plays a crucial role in facilitating tumor development and progression [1–5]. Peripheral blood counts of neutrophils, lymphocytes, and platelets, which reflect the inflammatory and immune status of the body, have been reported as potential prognostic factors for various types of cancers, when expressed as ratios [6–10]. In the context of cancer immunotherapy, the neutrophil-to-lymphocyte ratio (NLR) and platelet-to-lymphocyte ratio are promising predictive biomarkers for the response to immune checkpoint inhibitors (ICIs) in non-small cell lung cancer (NSCLC) [11]. Moreover, the systemic immune-inflammation index (SII), a novel integrated biomarker calculated as platelet count × neutrophil count/lymphocyte count (platelet count × NLR), demonstrates superior prognostic value along with NLR in hepatocellular carcinoma [12] and is associated with survival outcomes in patients with NSCLC treated with ICIs [13].

Small-cell lung cancer (SCLC) is a highly aggressive and lethal form of lung cancer with high metastatic potential and is characterized by rapid tumor growth, high vascularity, genomic instability, and early metastatic dissemination. It results in an estimated 250,000 deaths worldwide annually, with a low 5-year survival rate of 7%, and accounts for approximately 15% of all lung cancer cases [14–16]. The recent incorporation of ICIs, such as durvalumab and atezolizumab, into the standard-of-care treatment for extensive-stage SCLC (ES-SCLC) has led to significant improvements in overall survival (OS) [17, 18]. However, the identification of prognostic biomarkers for ICIs in SCLC remains an important research goal.

Despite growing evidence supporting the prognostic value of the SII in various malignancies, its relationship with survival outcomes in patients with SCLC treated with ICIs remains largely unexplored. In clinical practice, substantial heterogeneity in the response to ICI maintenance therapy has been observed in SCLC, with some patients achieving durable responses, whereas others experience early disease progression. Therefore, the identification of reliable prognostic biomarkers for ICIs in SCLC is imperative to better understand patient outcomes and facilitate risk stratification.

The present study aimed to evaluate the prognostic impact of the SII after the introduction of ICIs in patients with SCLC treated with chemoimmunotherapy followed by ICI maintenance therapy. We hypothesized that an elevated SII in the initial phase of ICI maintenance therapy is associated with inferior survival outcomes and could potentially serve as a readily available prognostic biomarker.

## Materials and methods

### Study design and patient population

This single-center retrospective study initially included 62 patients with histologically or cytologically confirmed SCLC who received chemoimmunotherapy at Yokohama Municipal Citizen’s Hospital between September 2019 and July 2024. Of these, 36 who subsequently received at least two cycles of ICI maintenance monotherapy were included in the final analysis. The eligibility criteria were treatment-naïve ES-SCLC and relapsed limited-stage SCLC (LS-SCLC), including post-surgical recurrence. Patients who received additional local therapies, such as radiotherapy, as deemed necessary by the treating clinicians, were included in the study cohort. The patients were administered platinum-based chemotherapy (a platinum agent plus etoposide) in combination with ICIs. The choice of the platinum agent and ICI was left to the discretion of the treating physicians based on the clinical characteristics and guidelines. The dose of cytotoxic agents was determined by the treating clinicians according to individual patient factors. Fixed doses of durvalumab (1500 mg/kg) and atezolizumab (1200 mg/kg) were administered for ICI therapy. The dosing interval for the ICI therapy was adjusted as required by the treating physician. In case of adverse events, ICI treatment was appropriately postponed or discontinued. Patients had to be clinically judged as having required systemic chemotherapy and received either durvalumab or atezolizumab as maintenance therapy. To be included in the analysis, patients had to complete a minimum of two cycles of ICI maintenance therapy. This criterion was established to ensure calculation of the SII at the start of the second maintenance cycle, thereby mitigating the potential confounding effects of cytotoxic chemotherapy-induced myelosuppression on hematological parameters. As the primary and central aim of this study was to investigate prognostic factors specifically for ICIs, we examined the SII solely during the maintenance phase of treatment when patients received ICI monotherapy to avoid the confounding influence of concurrent cytotoxic chemotherapy. Patients were stratified into high SII (≥ 570) and low SII (< 570) groups based on their SII values at the initiation of the second cycle of ICI maintenance to evaluate the prognostic value of the SII for survival outcomes.

### Data collection and assessment

Demographic and clinical data were collected from electronic medical records, including age at diagnosis, sex, smoking history, metastatic sites, peripheral blood counts, Eastern Cooperative Oncology Group performance status (ECOG PS), type of ICI, recurrence status, and whether patients received local therapy within the previous month of chemotherapy initiation. Peripheral blood platelet, neutrophil, and lymphocyte counts were determined from routine blood tests performed at the beginning of the second ICI maintenance cycle. Tumor response was assessed by investigators according to the Response Evaluation Criteria in Solid Tumors (RECIST) version 1.1. Imaging was performed using whole-body non-contrast or contrast-enhanced computed tomography, brain contrast-enhanced magnetic resonance imaging (MRI), or non-contrast MRI. Imaging assessments were scheduled at every two cycles of chemotherapy, with adjustments made at the discretion of the treating clinicians based on individual patient needs and clinical circumstances.

### Calculation of SII

**Fig. 1:**
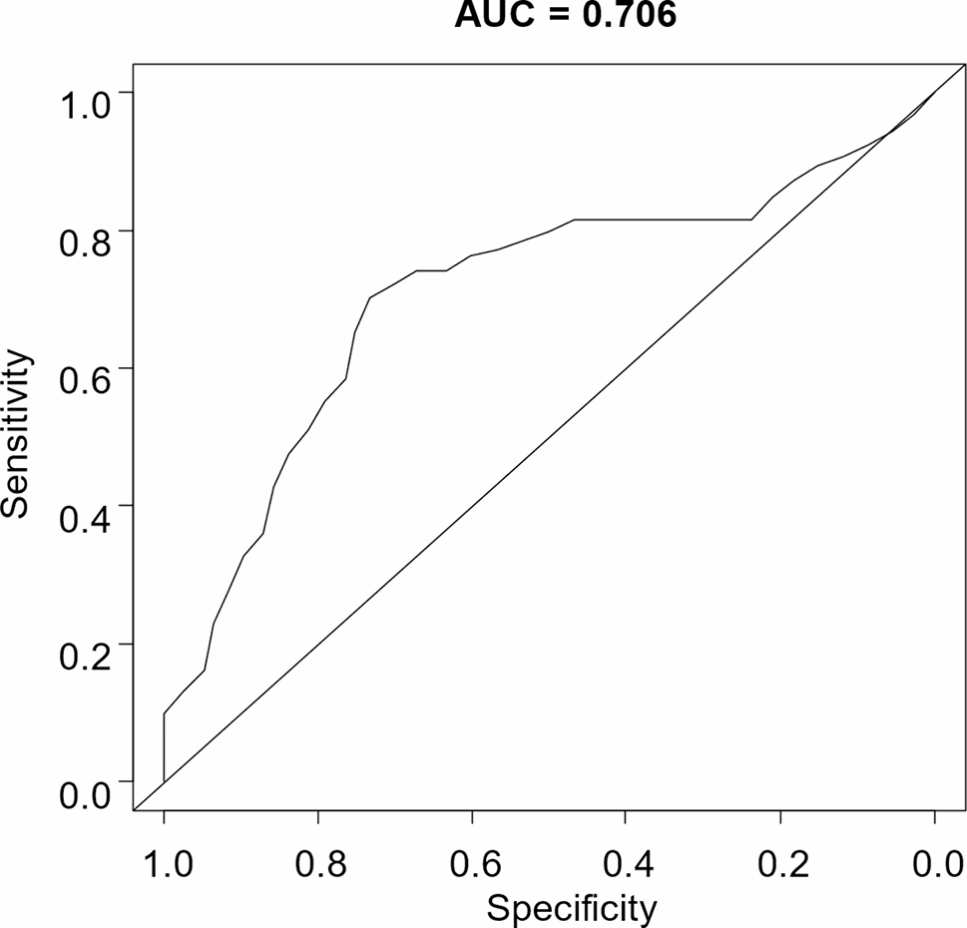
Receiver operating characteristic (ROC) curve analyses for survival duration. ROC curve analysis using a systemic immune-inflammation index (SII) cut-off value of 570

SII was calculated as platelet count (×10^3^/µL) × neutrophil count (/µL)/lymphocyte count (/µL). The optimal SII cutoff for OS was determined using receiver operating characteristic (ROC) curve analysis (Fig. 1), considering previously reported cutoffs [19].

For the ROC curve analysis of the SII with regard to OS, the evaluation time point was set at 13 months, corresponding to the median OS observed in the CASPIAN trial, which investigated durvalumab plus platinum-etoposide in ES-SCLC [17]. The analysis employed the nearest neighbor method with a span of 0.05. The resulting SII cut-off value was 570. The resulting area under the curve (AUC) was 0.706, indicating moderate accuracy. The optimal SII cut-off value of 570 derived from this analysis was deemed acceptable and aligned with the range reported previously [19].

This comprehensive approach to determine cut-off values ensures a robust foundation for the subsequent analysis of the prognostic significance of the SII in patients with SCLC receiving ICI maintenance therapy.

### Study endpoints and statistical analysis

The primary endpoint was the comparison of OS between high (≥ 570) and low (< 570) SII groups, with OS defined as the time from the start of combination immunotherapy until last follow-up or death from any cause. The secondary endpoint was the comparison of progression-free survival (PFS) between the SII groups, with PFS defined as the time from the start of combination immunotherapy until disease progression as per the RECIST version 1.1, death, or last follow-up. Kaplan–Meier survival curves were generated and compared using log-rank tests.

Multivariate analyses using the Cox proportional hazards model were performed to identify independent prognostic factors for both OS and PFS, with the number of variables determined based on an event-per-variable ratio of 5 [20]. Variables included in the models were age, sex, ECOG PS ≥ 2, presence of central nervous system (CNS) metastasis, and SII ≥ 570, selected based on their clinical relevance and potential impact on survival in SCLC. ECOG PS ≥ 2 and CNS metastasis are generally considered poor prognostic factors.

Before employing the log-rank test and Cox proportional hazards model, we verified the proportional hazards assumption for both OS and PFS. Different methods were employed to verify the proportional hazards assumption for each statistical analysis. For log-rank tests, the assumption was verified through visual inspection of log-log plots, which demonstrated approximately parallel lines between SII groups (≥ 570 vs. < 570). For Cox proportional hazards models, formal statistical testing using Schoenfeld residuals confirmed no significant violation of the proportional hazard’s assumption in both OS and PFS analyses.

Fisher’s exact test was used to compare categorical variables, and *t*-test was used to evaluate continuous variables. All tests were two-sided, and statistical significance was set at *P* < 0.05. Statistical analyses were performed using the EZR tool (Saitama Medical Center, Jichi Medical University), a modified version of the R command. EZR includes the common biostatistical functions used in R (R Foundation for Statistical Computing) [21].

### Ethical considerations

This study was conducted in accordance with the ethical principles outlined in the Declaration of Helsinki and was approved by the Institutional Review Board of Yokohama Municipal Citizen’s Hospital. Owing to the retrospective nature of this study and the use of anonymized data from existing medical records, the requirement for individual informed consent was waived. This waiver was granted based on the noninvasive nature of the study, use of only anonymized data with adequate measures to protect personal information, impracticality of contacting the study subjects, and minimal risk of infringing upon the rights or interests of the patients involved. All data were handled confidentially and in compliance with relevant privacy laws and regulations. The study protocol ensured that the research objectives could be achieved without compromising patient privacy or the ethical standards of medical research.

## Results

### Patient characteristics and baseline demographics

Patients were stratified into two groups: SII ≥ 570 (*n* = 14) and SII < 570 (*n* = 22). Table 1 provides a summary of the baseline characteristics and demographics. The median age was 74 years (range: 51–88 years) in the SII ≥ 570 group and 73 years (range: 56–87 years) in the SII < 570 group (*P* = 0.80). Males predominated in both groups, accounting for 85.7% of patients in the SII ≥ 570 group and 90.9% of those in the SII < 570 group (*P* = 0.63). The proportion of current or ex-smokers was similar across groups (92.9% in SII ≥ 570; 95.5% in SII < 570; *P* = 1.00). The ECOG PS distribution did not differ significantly between groups (PS ≥ 2: 35.7% in SII ≥ 570; 13.6% in SII < 570; *P* = 0.10). The distribution of ICI types was also similar (atezolizumab: 64.3% in SII ≥ 570; 40.9% in SII < 570; *P* = 0.31).

**Table 1:** Characteristics of participants (*n* = 36)

Characteristic	SII ≥ 570 group (*n* = 14)	SII < 570 group (*n* = 22)	*P*-value
Median age, years (range)	74 (51–88)	73 (56–87)	0.80
Sex			
Men	12 (85.7%)	20 (90.9%)	0.63
Women	2 (14.3%)	2 (9.1%)	
Smoking status			
Never	1 (7.1%)	1 (4.5%)	1
Current or Ex-smokers	13 (92.9%)	21(95.5%)	
ECOG performance status			
0	2 (14.3%)	2 (9.1%)	0.10
1	7 (50.0%)	17 (77.3%)	
2	2 (14.3%)	3 (13.6%)	
3	3 (21.4%)	0 (0%)	
4	0 (0%)	0 (0%)	
ICIs			
Atezolizumab	9 (64.3%)	9 (40.9%)	0.31
Durvalumab	5 (35.7%)	13 (59.1%)	
Site of metastasis			
Adrenal gland metastasis	2 (14.3%)	4 (18.1%)	1
Bone metastasis	7 (50.0%)	3 (13.6%)	0.026
Liver metastasis	6 (42.9%)	4 (18.2%)	0.14
CNS metastasis with unstable symptoms without symptoms	0 (0%) 1 (7.1%)	1 (4.5%) 3 (13.6%)	1 1
Recurrent cases	5 (35.7%)	7 (31.8%)	1
Cases receiving local therapy within 1 month before chemotherapy initiation	1 (7.1%)	1 (4.5%)	1

Abbreviations: SII, systemic immune-inflammation index; ECOG, Eastern Cooperative Oncology Group; ICIs, immune checkpoint inhibitors; CNS, central nervous system

With respect to metastatic sites, no statistically significant differences were observed in the frequencies of adrenal gland metastasis (14.3% in SII ≥ 570; 18.2% in SII < 570; *P* = 1.00), liver metastasis (42.9% in SII ≥ 570; 18.2% in SII < 570; *P* = 0.14), CNS metastasis with unstable symptoms (0% in SII ≥ 570; 4.5% in SII < 570; *P* = 1.00), or CNS metastasis without symptoms (7.1% in SII ≥ 570; 13.6% in SII < 570; *P* = 1.00). However, the frequency of bone metastasis was significantly higher in the SII ≥ 570 group than in the SII < 570 group (50.0% vs. 13.6%; *P* = 0.026). The proportion of recurrent cases was similar in the two groups (35.7% in SII ≥ 570; 31.8% in SII < 570; *P* = 1.00). Additionally, the number of patients receiving local therapy within the previous month of chemotherapy initiation was comparable (7.1% in SII ≥ 570; 4.5% in SII < 570; *P* = 1.00).

**Fig. 2:**
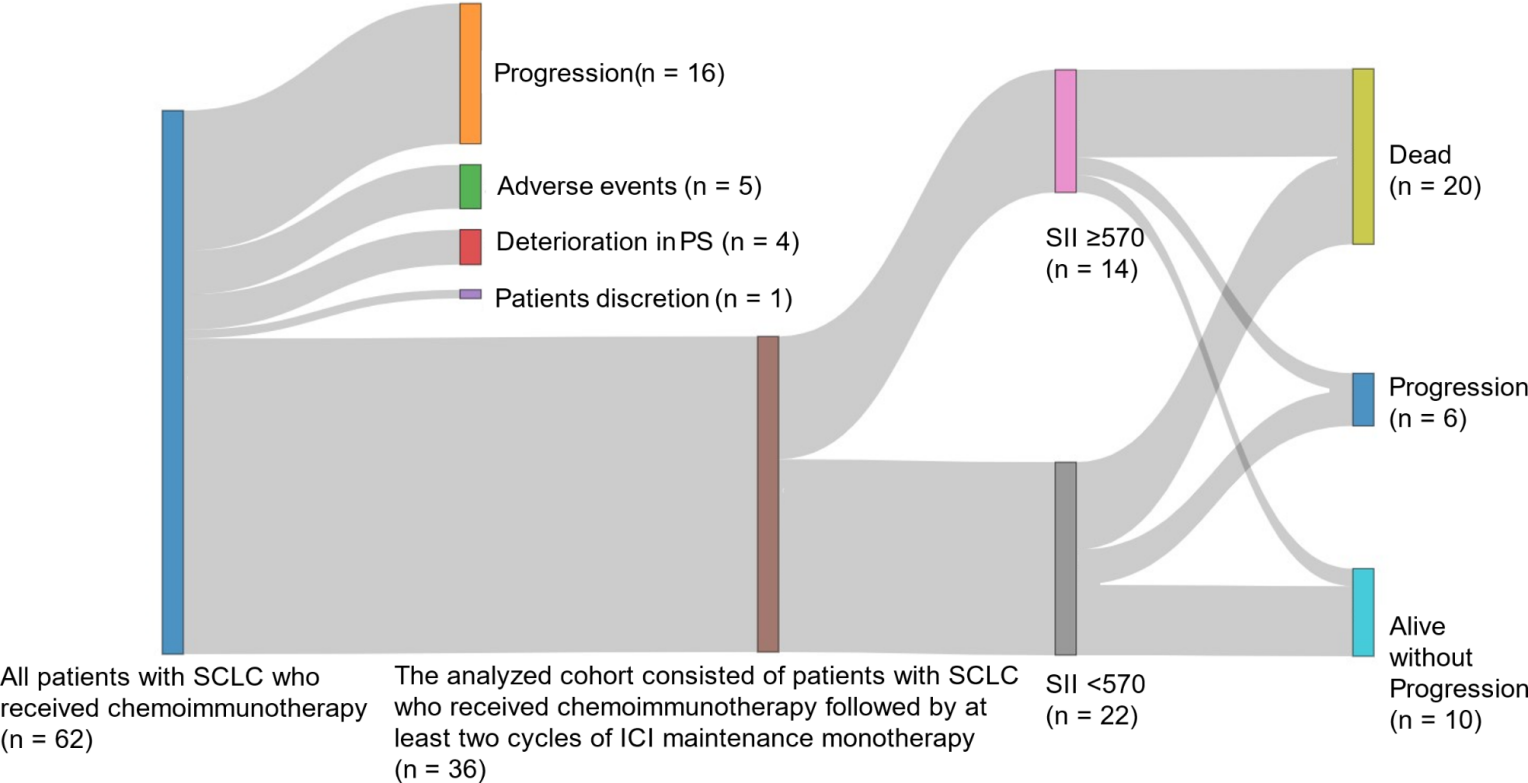
Sankey diagram: 62 patients with small cell lung cancer (SCLC) receiving chemoimmunotherapy. The first node represents the 62 patients who received chemoimmunotherapy. The second node shows the patients who were excluded from the analysis for various reasons, including disease progression (*n* = 16), adverse events (*n* = 5), deterioration in performance status (PS; *n* = 4), and patient discretion (*n* = 1). The third node displays the remaining patients (*n* = 36) who proceeded to receive at least two cycles of immune checkpoint inhibitor (ICI) maintenance monotherapy, and were included in the final analysis. The fourth node classifies patients based on their systemic immune inflammation index (SII): SII < 570 (*n* = 22) and SII ≥ 570 (*n* = 14). The final node presents the patient outcomes, indicating those who succumbed to the disease (*n* = 20), experienced disease progression (*n* = 6), or remained alive without disease progression (*n* = 10)

Figure 2 shows a Sankey diagram illustrating the flow of 62 patients with histologically or cytologically confirmed SCLC who received chemoimmunotherapy during each phase of the study. The first node represents the 62 patients who received chemoimmunotherapy. The second node shows the patients who were excluded from the analysis for various reasons, including disease progression (*n* = 16), adverse events (*n* = 5), PS deterioration (*n* = 4), and patient discretion (*n* = 1). The third node shows the remaining patients (*n* = 36) who received at least two cycles of ICI maintenance monotherapy and were included in the final analysis. The fourth node indicates classification of patients based on their SII: SII < 570 (*n* = 22) and SII ≥ 570 (*n* = 14). The final node presents the patient outcomes, indicating those who succumbed to the disease (*n* = 20), experienced disease progression (*n* = 6), or remained alive without disease progression (*n* = 10).

### Survival analysis and prognostic impact of SII

**Fig. 3:**
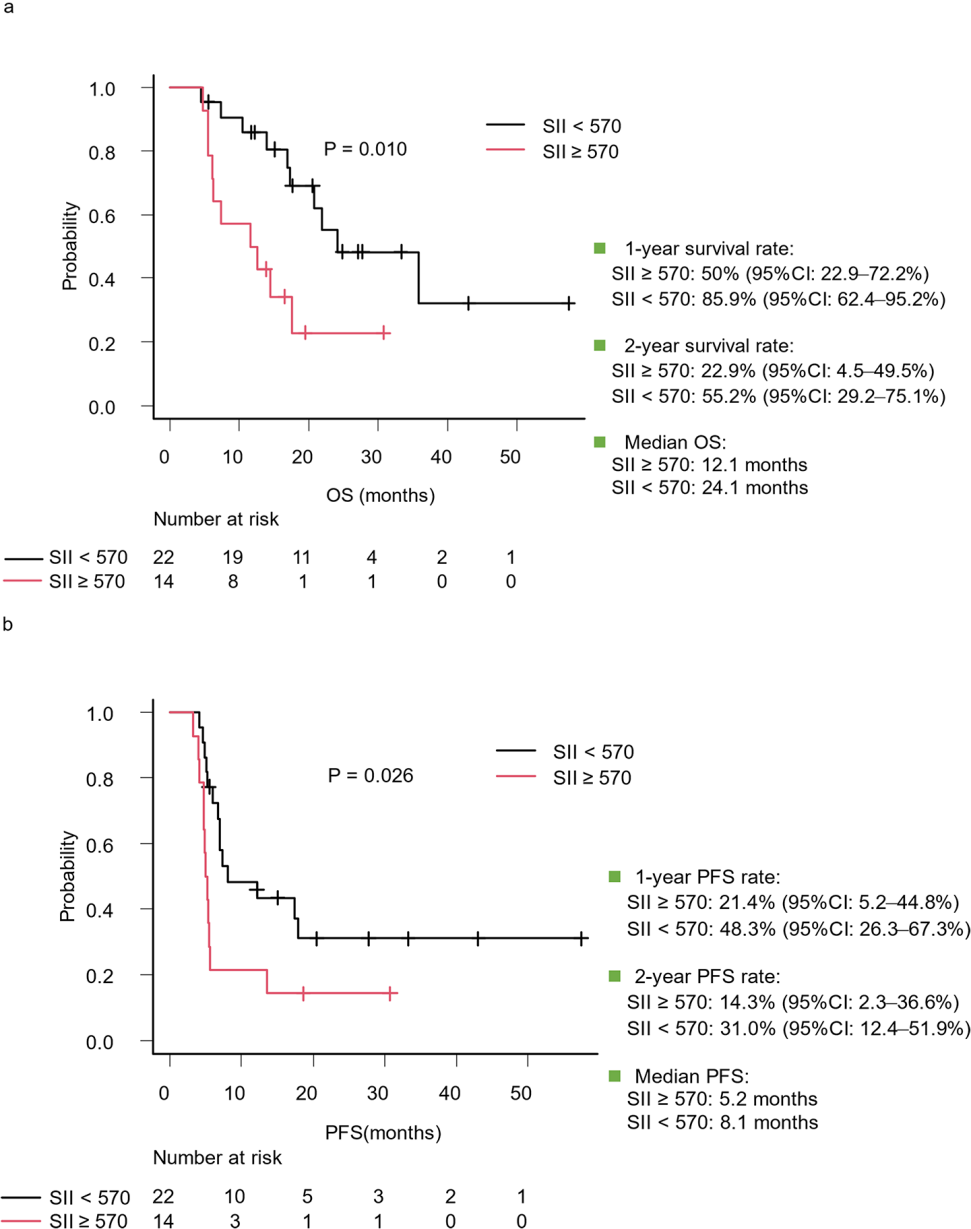
Kaplan–Meier survival analyses comparing outcomes based on systemic immune-inflammation index (SII) values. (**a)** Overall survival (OS) stratified by SII < 570 and SII ≥ 570. Patients with SII < 570 demonstrated a significantly improved OS compared with that of patients with SII ≥ 570 (median OS: 24.1 vs. 12.1 months, respectively; *P* = 0.010). 1-year survival rates were 85.9% (95% confidence interval [CI]: 62.4–95.2%) for SII < 570 and 50% (95% CI: 22.9–72.2%) for SII ≥ 570. 2-year survival rates were 55.2% (95% CI: 29.2–75.1%) for SII < 570 and 22.9% (95% CI: 4.5–49.5%) for SII ≥ 570. (**b**) Progression-free survival (PFS) stratified by SII < 570 and SII ≥ 570. Patients with SII < 570 exhibited significantly longer PFS compared with that of patients with SII ≥ 570 (median PFS: 8.1 vs. 5.2 months, respectively; *P* = 0.026). 1-year PFS rates were 48.3% (95% CI: 26.3–67.3%) for SII < 570 and 21.4% (95% CI: 5.2–44.8%) for SII ≥ 570. 2-year PFS rates were 31.0% (95% CI: 12.4–51.9%) for SII < 570 and 14.3% (95% CI: 2.3–36.6%) for SII ≥ 570

The median follow-up duration of the study cohort was 20.3 months. A Kaplan–Meier survival analysis with the log-rank test, as depicted in Fig. 3a, revealed a significantly shorter OS in the SII ≥ 570 group than in the SII < 570 group (*P* = 0.010). The median OS was 12.1 months in the SII ≥ 570 group compared with 24.1 months in the SII < 570 group. Estimated 1-year OS rates derived from the Kaplan–Meier curves were 50.0% (95% CI: 22.9–72.2%) in the SII ≥ 570 group and 85.9% (95% CI: 62.4–95.2%) in the SII < 570 group. Furthermore, the 2-year OS rates were 22.9% (95% CI: 4.5–49.5%) in the SII ≥ 570 group and 55.2% (95% CI: 29.2–75.1%) in the SII < 570 group.

An exploratory PFS analysis using the Kaplan–Meier survival analysis and the log-rank test, illustrated in Fig. 3b, also demonstrated a significantly shorter PFS in the SII ≥ 570 group than in the SII < 570 group (*P* = 0.026). The median PFS was 5.2 months in the SII ≥ 570 group versus 8.1 months in the SII < 570 group. In the Kaplan–Meier analysis, the estimated 1-year PFS rate for the SII ≥ 570 group was 21.4% (95% CI: 5.2–44.8%), whereas that for the SII < 570 group was 48.3% (95% CI: 26.3–67.3%). The analysis further revealed 2-year PFS rates of 14.3% (95% CI: 2.3–36.6%) for the SII ≥ 570 group and 31.0% (95% CI: 12.4–51.9%) for the SII < 570 group.

### Multivariate analysis of prognostic factors

Multivariate analysis using the Cox proportional hazards model was performed to identify the independent prognostic factors for OS (Table 2a) and PFS (Table 2b). After adjusting for potential confounders, including age, sex, PS ≥ 2, and presence of CNS metastasis, SII ≥ 570 was identified as an independent negative prognostic factor for both OS (HR: 3.83, 95% CI: 1.38–10.6, *P* = 0.010) and PFS (HR: 2.66, 95% CI: 1.11–6.33, *P* = 0.028).

For OS, age (Hazard Ratio [HR]: 0.977 per year increase; 95% CI: 0.924–1.03; *P* = 0.41), male sex (HR: 2.27 compared with female sex; 95% CI: 0.422–12.2; *P* = 0.34), ECOG PS ≥ 2 (HR: 1.43 compared with ECOG PS < 2; 95% CI: 0.374–5.44; *P* = 0.60), and presence of CNS metastasis (HR: 2.04 compared with the absence of CNS metastasis; 95% CI: 0.553–7.54; *P* = 0.28) were not significantly associated with OS in the multivariate model.

Similarly, for PFS, age (HR: 1.00 per year increase; 95% CI: 0.955–1.05; *P* = 0.92), male sex (HR: 2.44 compared with female sex; 95% CI: 0.511–11.6; *P* = 0.26), ECOG PS ≥ 2 (HR: 1.37 compared with ECOG PS < 2; 95% CI: 0.470–4.02; *P* = 0.56), and presence of CNS metastasis (HR: 2.62 compared with the absence of CNS metastasis; 95% CI: 0.800–8.60; *P* = 0.11) were not significantly associated with PFS in the multivariate model.

**Table 2a:** Multivariate analysis using a Cox proportional hazard regression model for OS

Factor	Hazard ratio (95% CI)	*P*-value
Age	0.977 (0.924–1.03)	0.41
Male (ref: Female)	2.27 (0.422–12.2)	0.34
PS ≥ 2 (ref: PS < 2)	1.43 (0.374–5.44)	0.60
CNS metastasis positive (ref: negative)	2.04 (0.553–7.54)	0.28
SII ≥ 570 (ref: SII < 570)	3.83 (1.38–10.6)	0.010

Abbreviations: CI, confidence interval; ref, reference; PS, performance status; CNS, central nervous system; SII, systemic immune inflammation index; OS, overall survival

**Table 2b:** Multivariate analysis using a Cox proportional hazard regression model for PFS

Factor	Hazard ratio (95% CI)	*P*-value
Age	1.00 (0.955–1.05)	0.92
Male (ref: Female)	2.44 (0.511–11.6)	0.26
PS ≥ 2 (ref: PS < 2)	1.37 (0.470–4.02)	0.56
CNS metastasis positive (ref: negative)	2.62 (0.800–8.60)	0.11
SII ≥ 570 (ref: SII < 570)	2.66 (1.11–6.33)	0.028

Abbreviations: CI, confidence interval; ref, reference; PS, performance status; CNS, central nervous system; SII, systemic immune inflammation index; PFS, progression-free survival

## Discussion

This study evaluated the prognostic significance of the SII measured during the initial phase of ICI maintenance therapy in patients with SCLC who received chemoimmunotherapy followed by ICI maintenance treatment. An elevated SII (≥ 570) was associated with a significantly shorter OS and PFS than a low SII (< 570). Furthermore, high SII was identified as an independent negative prognostic factor for OS and PFS. These results suggest that the SII, a readily obtained biomarker derived from routine blood tests, could potentially serve as a valuable tool for risk stratification and prognostication in patients with SCLC treated with ICIs, expanding the clinical applications of the SII. To the best of our knowledge, this study provides the first evidence of the prognostic significance of the SII in the initial phase of ICI maintenance therapy in patients with SCLC receiving ICI treatment.

Several studies have documented the efficacy of the NLR as a prognostic factor in patients with various malignancies [8–11]. Considering the prognostic factors for patients treated with ICIs, SII, which incorporates the platelet count by multiplying it with the NLR, may theoretically offer additional utility as a prognostic indicator. The potential benefit of the SII is attributed to the integration of the platelet component into the assessment. The inferior survival outcomes observed in patients with a high SII may be attributed to the immunosuppressive effects of platelets and their complex interactions with the tumor microenvironment. Platelets secrete various growth factors and cytokines, such as vascular endothelial growth factor (VEGF) and transforming growth factor-beta (TGF-β), which can promote tumor growth, angiogenesis, and immune evasion [22, 23]. VEGF overexpression has been associated with the suppression of immune effector cells and the activation of immunosuppressive cells [24]. Additionally, TGF-β plays a crucial role in immune suppression by inhibiting cytotoxic T cells, promoting regulatory T cell induction, and facilitating myeloid-derived suppressor cell differentiation [25–27]. Collectively, these findings suggest that platelets influence the immune landscape within the tumor microenvironment significantly, potentially modulating ICI efficacy.

In ovarian cancer, an increased platelet count is associated with disease progression and shortened survival. Regarding the mechanism underlying this relationship, in response to the overproduction of tumor-derived IL-6, the hepatic synthesis of thrombopoietin increases, inducing secondary thrombocytosis. The resultant elevated platelet count is thought to promote tumor growth by influencing the tumor microenvironment through processes such as tumor microvessel stabilization and pericyte development, thereby establishing a positive feedback loop [28].

Secondary thrombocytosis in the peripheral blood arises owing to tumor progression, and the increased platelets may again form a positive feedback loop with the tumor microenvironment while influencing the immune environment. Evidently, the peripheral blood platelet count plays a significant role in predicting the prognosis of patients undergoing ICI therapy. Therefore, the SII, which incorporates the platelet count in addition to the NLR, is a useful prognostic marker.

The clinical implications of our findings lie in the potential use of the SII as a prognostic tool to inform the timing and frequency of follow-up evaluations in patients with SCLC receiving ICI therapy. Patients with a high SII during the initial phase of ICI maintenance therapy may benefit from closer monitoring to facilitate early detection of disease progression and timely initiation of subsequent therapies. The simplicity and accessibility of the SII make it an attractive biomarker for clinical use. However, the optimal cut-off value remains to be definitively established. Nonetheless, persistently elevated neutrophil and platelet counts along with low lymphocyte counts during the initial phase of ICI maintenance therapy (i.e., a failure to achieve “hematological remission” following induction chemotherapy) should raise concerns about a poor prognosis.

This study has certain limitations, including its retrospective, single-center design and relatively small sample size, which may introduce potential bias. The small sample size can be attributed to real-world clinical challenges, as illustrated in the Sankey diagram, in which a significant proportion of patients fail to reach the maintenance therapy stage owing to adverse events, PS deterioration, or disease progression. This finding highlights a lesser-known aspect of the treatment journey for SCLC, whereby the attrition rate from induction chemoimmunotherapy to ICI maintenance therapy is considerable, reflecting the practical hurdles encountered in the management of this aggressive malignancy. This sample size limitation also affected our statistical approach in an important way. While our analysis identified a higher prevalence of bone metastasis in the high SII group (50.0% vs. 13.6%, *P* = 0.026), several methodological considerations prevented its inclusion in our multivariate analysis. Our statistical approach was guided by the established principle of maintaining an event-per-variable ratio of 5 to prevent model overfitting, as recommended by Vittinghoff and McCulloch [20]. Our multivariate model combines four well-established prognostic factors in SCLC (age, sex, ECOG PS ≥ 2, and CNS metastasis) that have been validated, together with SII ≥ 570, our proposed prognostic biomarker under investigation. These established variables serve as essential controls that should be retained in the model. Although the significant difference in bone metastasis distribution between SII groups is noteworthy, particularly in light of recent evidence demonstrating bone metastasis as a poor prognostic factor in ES-SCLC patients receiving chemoimmunotherapy [29], adding it as an additional variable would exceed our predetermined statistical constraints and potentially compromise the model’s reliability through overfitting. The interplay between bone metastasis and SII warrants further investigation in larger cohorts where additional variables can be incorporated into the multivariate model without risking statistical overfit. A second major limitation concerns the heterogeneity in our treatment settings. Heterogeneity in the treatment settings may have confounded the results. Notably, our cohort included a diverse range of SCLC patients, including those with ES-SCLC receiving first-line treatment, those with LS-SCLC experiencing recurrence (including postsurgical recurrence), and those who underwent additional local therapy during their treatment course. This heterogeneity in disease stage and treatment modalities could potentially affect the interpretation of our findings and limit their generalizability to specific SCLC subpopulations. An additional methodological limitation is that the optimal cut-off value for the SII has not been definitively established across various cancer types, including NSCLC, SCLC, and hepatocellular carcinoma [12, 13, 19]. This lack of standardization in SII cut-off values across different malignancies underscores the need for careful interpretation and validation of our findings. Prospective validation in larger, multi-institutional cohorts with more homogeneous patient populations is necessary to confirm the prognostic value of the SII in patients with SCLC treated with ICIs and to establish a standardized cut-off value for this specific clinical context. Despite these limitations, our study provides valuable real-world data for the use of the SII as a prognostic marker in patients with SCLC treated with chemoimmunotherapy. Real-world evidence can offer insights into the practical applications and effectiveness of biomarkers in diverse clinical settings, thus complementing the findings of tightly controlled clinical trials. Although limited in some respects, the heterogeneity of our patient population reflects the complexity of SCLC management in clinical practice. Therefore, our findings may serve as a useful reference for clinicians dealing with similarly diverse SCLC patient populations and could aid in the design of future prospective studies aimed at validating the prognostic utility of the SII in more specific SCLC subgroups.

## Conclusion

An elevated SII during the initial phase of ICI maintenance therapy was associated with an inferior OS and PFS in patients with SCLC who received chemoimmunotherapy followed by ICI maintenance. The SII, an accessible biomarker derived from routine blood tests, may improve risk stratification and prognostication in this patient population. Prospective studies are warranted to validate these findings and further elucidate the optimal cut-off value for the SII to guide clinical decision-making. Nonetheless, our study provides novel insights into the prognostic significance of the SII in patients with SCLC treated with ICIs and highlights the potential of this biomarker to aid treatment strategies and surveillance approaches in the field of cancer immunotherapy.

## Data Availability

The data that support the findings of this study are not openly available due to privacy and ethical restrictions, but are available from the corresponding author upon reasonable request. Data are located in controlled access data storage at Yokohama Municipal Citizen’s Hospital.
